# Characterization of a novel model for atherosclerosis imaging: the apolipoprotein E-deficient rat

**DOI:** 10.1186/s13550-023-01055-5

**Published:** 2023-12-11

**Authors:** Jürgen W. A. Sijbesma, Aren van Waarde, Sebastiaan Kristensen, Ilse Kion, Uwe J. F. Tietge, Jan-Luuk Hillebrands, Marian L. C. Bulthuis, Hendrik Buikema, Dalibor Nakladal, Marit Westerterp, Fan Liu, Hendrikus H. Boersma, Rudi A. J. O. Dierckx, Riemer H. J. A. Slart

**Affiliations:** 1 Department of Nuclear Medicine and Molecular Imaging, University Medical Center Groningen, University of Groningen, Hanzeplein 1, 9713 GZ Groningen, The Netherlands; 2 Department of Electrical and Computer Engineering, Aarhus University, Aarhus, Denmark; 3 Division of Clinical Chemistry, Department of Laboratory Medicine, Karolinska Institutet, Stockholm, Sweden; 4 Clinical Chemistry, Karolinska University Laboratory, Karolinska University Hospital, Stockholm, Sweden; 5 Division of Pathology, Department of Pathology and Medical Biology, University of Groningen, University Medical Center Groningen, Groningen, The Netherlands; 6 Department of Clinical Pharmacy and Pharmacology, University Medical Center Groningen, University of Groningen, Groningen, The Netherlands; 7 Comenius University Science Park, Bratislava, Slovakia; 8 5th Department of Internal Medicine, Faculty of Medicine, Comenius University Bratislava, Bratislava, Slovakia; 9 Department of Pediatrics, University Medical Center Groningen, University of Groningen, Groningen, The Netherlands; 10 Faculty of Science and Technology, Department of Biomedical Photonic Imaging, University of Twente, Enschede, The Netherlands

**Keywords:** Apolipoprotein E-deficient rat, Atherosclerosis, [^18^F]2-fluoro-2-deoxy-D-glucose, Positron emission tomography (PET)

## Abstract

**Background:**

The apolipoprotein E-deficient (*apoE*^*−/−*^) mouse is a well-established model for studying atherosclerosis. However, its small size limits its use in longitudinal positron emission tomography (PET) imaging studies. Recently, the *apoE*^*−/−*^ rat has emerged as an alternative. With this study, we investigate the feasibility of using *apoE*^*−/−*^ rats as an in vivo model for longitudinal atherosclerotic PET/CT imaging.

**Results:**

*ApoE*^*−/−*^ rats showed significantly higher [^18^F]FDG uptake than controls in the aortic arch (+ 18.5%, *p* < 0.001) and abdominal aorta (+ 31.0%, *p* < 0.001) at weeks 12, 26, and 51. *ApoE*^*−/−*^ rats exhibited hypercholesterolemia, as evidenced by plasma cholesterol levels that were up to tenfold higher, and total hepatic cholesterol levels that were up to threefold higher than the control rats at the end of the study. Fast protein liquid chromatography cholesterol profiling indicated very high levels of pro-atherogenic apoB-containing very low-density lipoprotein and low-density lipoprotein fractions in the *apoE*^*−/−*^ rats. Atherosclerotic lesions cover 19.9% of the surface of the aortic arch (*p* = 0.0013), and there was a significantly higher subendothelial accumulation of ED1-positive macrophages in the abdominal aorta of the *apoE*^*−/−*^ rats compared to control rats (Ctrl) (*p* = 0.01). No differences in neutral sterols were observed but higher levels of bile acids were found in the *apoE*^*−/−*^ rats.

**Conclusion:**

These data demonstrate early signs of hypercholesterolemia, high levels of bile acids, the development of atherosclerotic lesions, and macrophage accumulation in *apoE*^*−/−*^ rats. Therefore, this model shows promise for atherosclerosis imaging studies.

**Supplementary Information:**

The online version contains supplementary material available at 10.1186/s13550-023-01055-5.

## Background

Genetically modified mouse models play a crucial role in the study and understanding of the mechanisms underlying atherogenesis. Among these models, in biomedical research, the apolipoprotein E-deficient (*apoE*^*−/−*^) mouse is widely recognized as one of the most commonly used preclinical models for studying plaque formation and progression [1]. ApoE acts as a ligand for receptors involved in the clearance of chylomicrons and remnants of very low-density lipoproteins (VLDL) [1, 2]. In *apoE*^*−/−*^ mice, the absence of apoE results in impaired lipoprotein clearance, leading to elevated plasma cholesterol levels. This elevation stimulates atherogenesis, resulting in the development of complex lesions similar to those found in humans. These lesions consist of a fibrous cap containing smooth muscle cells, foam cells (lipid-loaden macrophages), and a necrotic core [3]. To accelerate the formation of lesions, mice are often fed a Western or high-fat diet, which initiates progressive plaque formation starting at the age of 10–12 weeks [4].

Positron emission tomography (PET) is an in vivo imaging technique that provides valuable insights into atherosclerotic processes in small animals. It involves labeling biomolecules such as proteins, metabolites, hormones, and drugs with a positron emitter, enabling their use as tracers to target specific biological processes. The metabolic PET tracer [^18^F]-fluoro-2-deoxy-D-glucose ([^18^F]FDG) is a radiolabeled glucose analog that is taken up by cells metabolizing glucose, including inflammatory cells involved in the atherosclerotic process, such as activated macrophages. Several clinical and preclinical studies on atherosclerosis have shown that the accumulation of [^18^F]FDG correlates with regions of high macrophage density in atherosclerotic vessels and plaques [5–8].

The combination of the *apoE*^−/−^ mouse model and [^18^F]FDG PET imaging presents new research opportunities but also poses challenges. Atherosclerotic lesions in *apoE*^−/−^ mice are often smaller than the resolution of a dedicated small animal PET system (1–1.5 mm), making it difficult to distinguish lesions from surrounding tissues due to partial volume effects [9].

Small animal PET imaging typically requires the use of anesthesia to prevent animal movement and motion artifacts. The combination of anesthesia and the high skin-surface-to-body-weight ratio of mice can lead to hypothermia [10]. Hypothermia can activate brown adipose tissue (BAT), resulting in increased [^18^F]FDG uptake, which can blur the [^18^F]FDG signal from atherosclerotic lesions [11–13].

Recently, an *apoE*^−/−^ rat model has become available, showing comparable characteristics to *apoE*^−/−^ mice but with the advantage of a larger size [14–16], which allows for easier detection of small structures such as blood vessels and atherosclerotic tissue. Although hypothermia and activation of BAT can still be a concern in rats, the impact is, due to a lower skin-surface-to-body-weight ratio, less significant compared to mice.

In this study, we aim to further characterize the *apoE*^−/−^ rat model and investigate its feasibility as an in vivo model for atherosclerosis (PET/CT) imaging.

## Methods

### Animals

Ten male apolipoprotein E-deficient rats (SD-ApoE^tm1sage^) (*apoE*^−/−^) from different litters, with a Sprague Dawley background and ten male Sprague Dawley wild-type control rats (Ctrl) at the age of 10 ± 1 weeks were obtained from Sage Labs Inc. located in Boyertown, Pennsylvania, U.S.A. Upon arrival, the rats were allowed a minimum acclimation period of 14 days to recover from transportation and adjust to their new housing conditions.

The rats were housed in Makrolon cages at a constant temperature of 21 ± 2 °C and maintained on a 12-h light/12-h dark cycle. Prior to the baseline scan, all animals were provided with a standard chow diet. Following the baseline scan, the rats were given ad libitum access to a Western diet high in fat (21%) and cholesterol (0.21%) (D12079B, Research Diets Inc., New Brunswick, New Jersey, U.S.A.). Additional details regarding the diet can be found in the Additional file 1: Table S1.

The experimental protocol was conducted in accordance with the ARRIVE guidelines and approved by the Central Committee on Animal Experiments of The Netherlands (license number AVD 105002016707) and the animal welfare body of the University Medical Center Groningen (protocol 16707-01-001).

### Experimental design and procedure

At the baseline assessment, non-fasted rats were anesthetized using isoflurane mixed with oxygen (5% for induction and 2% for maintenance). A blood sample for biochemistry measurements and plasma glucose levels was collected from the tail vein. Subsequently, a bolus injection of approximately 64 ± 5 MBq of [^18^F]FDG was administered intravenously. After the injection, the rats were placed back in a pre-heated home cage to recover.

To optimize the contrast between plaque and background [17–19], 3 h were allowed to elapse post-injection before the rats were re-anesthetized and positioned in a dedicated small animal PET/CT camera (D-PET, Inveon®, Siemens Preclinical Solutions, Knoxville, Tennessee, U.S.A.). The rats were positioned with their thorax at the center of the field of view. A CT scan was performed using the following parameters: 40 kV, 250 µA, total rotation of 360° in 360 steps, and an exposure time of 350 ms. Subsequently, a 20-min static PET scan was conducted.

During the PET scan, the animals were kept warm using heating pads and a thermostat set to a temperature of 38 °C (M2M Imaging, Cleveland, Ohio, U.S.A.). After the baseline scan, the rats were returned to a pre-heated home cage to recover from the procedure.

The entire procedure was repeated at weeks 4, 12, 26, and 51 ± 1 after the baseline assessment. Body weight was measured weekly (EL2000 scale, Shimadzu, Kyoto, Japan). Following the final scan, the rats were euthanized, and the heart and vessels were perfused using saline. The aortic arch, abdominal aorta, liver, cecum content, and faeces were collected and stored for further analysis.

### PET and CT analysis

The PET data obtained from the scans were reconstructed using an ordered set expectation maximization-3D/maximum a posteriori (OSEM3D/MAP) algorithm. The reconstruction process involved 2 OSEM iterations followed by 18 MAP iterations. The final reconstructed PET datasets had an in-plane image matrix size of 256 × 256 pixels, with a voxel size of 0.388 × 0.388 × 0.796 mm and a resolution of 1.5 mm at the center of the field-of-view. The scans were corrected for decay, random coincidences, scatter, and attenuation.

The CT data were reconstructed using the Filtered Back Projection (FBP) algorithm. A low noise reduction technique, a Shepp-Logan filter, and a beam hardening correction were applied during the reconstruction process. The resulting CT images had a maximum voxel size of 0.099 × 0.099 × 0.099 mm and a pixel size of 99.24 µm.

To fuse the PET and CT images, the PMOD version 3.9 software (PMOD Technologies Ltd., Zürich, Switzerland) was used. A 20 × 20 × 20 mm box was created and positioned rostrally, immediately above the heart, to include the aortic arch within the box. Voxels outside of the box were masked, and background noise was eliminated by excluding voxels with values below 60 kBq/cc. For the abdominal aorta, a 15 × 15 × 15 mm box was created and positioned in line with the diaphragm. Similar to the aortic arch, voxels outside of the box were masked, and background noise within the box was removed by excluding voxels with values below 60 kBq/cc. Subsequently, the average and the maximum standardized uptake value (SUV_mean_, SUV_max_) of [^18^F]FDG was calculated in the tissue in the selected boxes Additionally, SUV_mean_ was corrected for plasma glucose levels using the following formula:SUV_corr_ = ((SUV_mean_ * plasma glucose level) / plasma glucose level_mean per group per time point_).

Extra regions of interest (ROI) were drawn, using the CT as anatomical reference, around the liver, lungs, kidneys, intestines, myocardium, vena cava and blood pool. Target-to-background ratio (TBR), defined as SUV_max_ aorta/ SUV_mean_ vena cava, was calculated for the aortic arch and abdominal aorta.

The percentage of body fat was determined using procedures described in the previous work [20].

### Lipid, lipoprotein, and bile acid analysis

During the study, blood samples were collected from the tail vein of the rats. A drop of blood was used to measure the plasma glucose levels (Accu-Chek Roche Mannheim, Germany). The rest of the plama was stored in EDTA-coated tubes. Total plasma cholesterol and triglycerides were measured using commercially available kits from Roche Diagnostics (Basel, Switzerland).

To analyze the lipoprotein subspecies, the plasma pool of each group was injected onto a Superose 6HR10/300GL column (GE Health, Uppsala, Sweden). The column utilized gel filtration to separate the different lipoprotein subspecies, following a previously described method [21]. At the time of animal euthanasia, the livers were excised and frozen in liquid nitrogen. For lipid analysis, lipids were extracted from liver homogenates using a modified version of the Bligh and Dyer procedure, as previously published [22]. The extracted lipids were then dissolved in water containing 2% Triton X-100. Hepatic total cholesterol and triglyceride levels were measured using commercially available reagents from Roche Diagnostics (Basel, Switzerland).

After collection at the end of the study fecal samples from the cecum were dried, weighed, and grinded thoroughly. For neutral sterol and bile acid extraction, 50 mg of grinded faeces was heated at 80 °C in alkaline methanol for 2 h, followed by extraction with petroleum ether according to previously published methods [23]. Bile acids were methylated using a mixture of acetyl chloride, trimethylsilytate with pyridine, N,O-bis(trimethyllysilyl)trifluoroacetamide, and trimethylchlorosilane. Gas–liquid chromatography was then used to measure the levels of fecal neutral sterols and bile acids, as detailed in a previous publication [23].

### Immunohistochemistry

To confirm the presence of atherosclerotic lesions, Oil Red O staining (ORO) for lipid accumulation was performed on the complete aortic arches using the *en face* procedure. Aortic arches were thawed and washed with water and then dehydrated using a 60% 1,2-propanediol solution. The dehydration process was repeated twice. Each aorta was incubated with ORO staining solution from Lifeline Cell Technology (Walkersville, Maryland, USA) for 10 min. After incubation, the aortas were washed with water. The unfolded arches were imaged using a Canon 200D digital camera with a Canon Macro EF-S 60 mm lens (Canon Inc., Tokyo, Japan). The obtained images were analyzed using ImageJ image analysis software (version 1.53i, U.S. National Institutes of Health, Bethesda, Maryland, U.S.A.). The area of Oil Red O-positive staining was divided by the total area to quantify the extent of staining.

To assess monocytes and macrophages, ED1 staining was performed on frozen abdominal aortas. ED1 is expressed by macrophages and monocytes. The 3 µm thick aorta sections were mounted on glass slides. The sections were fixed with acetone and washed with PBS. Endogenous peroxidase activity was blocked using 0.009% H_2_O_2_. The sections were then incubated for 60 min at room temperature with anti-ED1 antibody (MCA341R, Bio-Rad Laboratories, Veenendaal, The Netherlands) diluted in PBS with 1%. After several washes with PBS, the sections were incubated with a secondary antibody (RAMPO, P0260, Dako, Amstelveen, The Netherlands) diluted in PBS with 1% BSA and 1% normal rat serum. Subsequently, the sections were incubated with a tertiary antibody (GARPO, P0448, Dako) diluted in PBS with 1% BSA and 1% normal rat serum. Peroxidase activity was visualized using the chromogen AEC (3-amino-9-ethylcarbazole), and the sections were counterstained with hematoxylin. The stained slides were imaged using a slide scanner (Hamamatsu NanoZoomer 2.0 HT, Iwata, Japan). The quantification of ED1-positive macrophages was performed using Aperio ImageScope software (version v12.4.3.5008, Leica Biosystems Imaging Inc., Vista, CA, U.S.A.). The number of strongly positive pixels was divided by the total surface area to quantify the presence of ED1-positive macrophages (N_sp_/A_tot_) [20] (algorithm is shown in Additional file 2: Table S2.)

### Statistical analysis

For repeated measurements, the Generalized Estimating Equations (GEE) model was used to account for missing data at the different time points in the design. The independent correlation matrix was selected for the analysis, and the Wald test was used to report *p*-values which were considered statistically significant at *p* < 0.05 without correction for multiple comparisons. For data with a single time point, a T-test was used to compare groups or conditions.

## Results

### General condition of the *apoE*^−/−^ rats

Throughout the study, no differences between the two strains were observed in the general condition of the rats, including their appearance, movement, drinking, and eating.

During the course of the experiment, there were a few cases of health issues among the rats. One Ctrl rat developed tumor growth in the abdomen within six months. Among the *apoE*^−/−^ rats, three rats died during the study. One rat had tumor growth, one rat died for unknown reasons, and one rat had an adverse reaction to anesthesia during a scan. Consequently, four rats did not complete the study.

Additionally, two *apoE*^−/−^ rats exhibited early signs of bumblefoot or pododermatitis, characterized by swelling in their feet [24], around halfway through the study. The animals did not display signs of discomfort. Importantly, the swelling did not progress further during the remainder of the study period.

### Different growth curves, body composition and plasma glucose levels

**Fig. 1:**
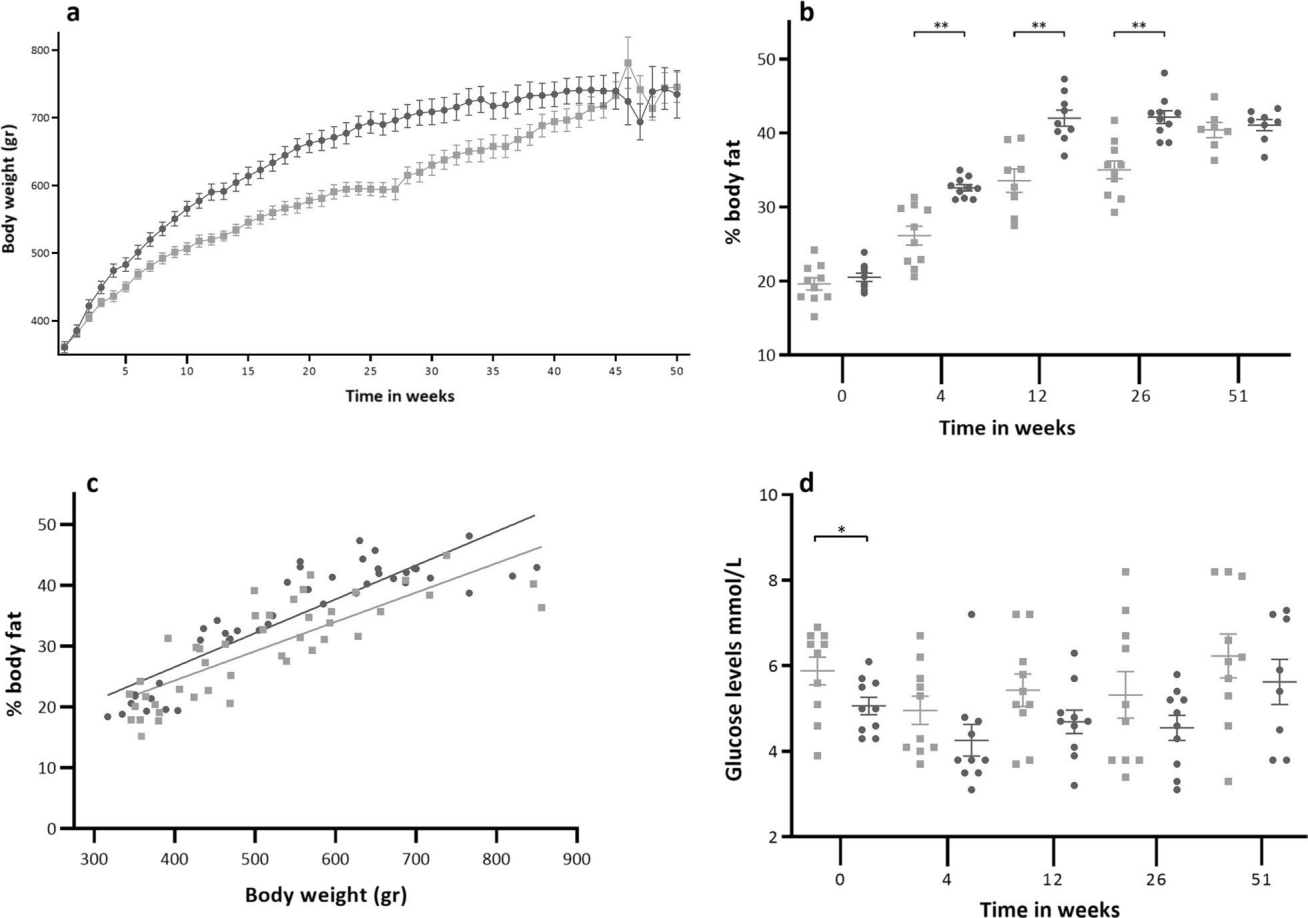
**a** Body weight in grams per strain per week. **b** % of body fat per strain per time point. **c** Correlation between body weight and % of body fat per group. Ctrl (*r* = 0.81, *p* < 0.0001), *apoE*^*−*^*/*^*−*^(*r* = 0.88, *p* < 0.0001). **d** Plasma glucose levels in mmol/L per strain per time point. Filled square Ctrl filled circle *ApoE*^−/−^. Data are presented as mean ± SEM.* is considered significantly different with a *p*-value < 0.05 and ** significantly different with a *p*-value < 0.001

Body weight at baseline was equal for both strains, with an average of 361 ± 4 g (Fig. 1a). After starting the Western diet, body weight increased in both groups, but the increase was more pronounced in the *apoE*^−/−^ rats. From week 3 to week 39, the average body weight of the *apoE*^−/−^ group was significantly higher than the Ctrl group, with a maximum difference of 102 g at week 27. After week 39, no significant differences in body weight were observed.

The percentage of body fat (Fig. 1b) followed a similar pattern as body weight, but the *apoE*^−/−^ rats reached their maximum percentage of body fat much earlier (week 12) compared to the Ctrl rats (week 51). Significant differences in body fat percentage between the two strains were observed at weeks 4 (*p* < 0.001), 12 (*p* < 0.001), and 26 (*p* < 0.001). At baseline (20%) and at the end of the study (40%), no significant differences in the percentage of body fat were found.

Body weight and the percentage of body fat showed (Fig. 1c) a significant correlation in both the Ctrl (*r* = 0.81, *p* < 0.001) and the *apoE*^−/−^ rats (*r* = 0.88, *p* < 0.001).

Overall, plasma glucose levels (Fig. 1d) were significantly lower in the *apoE*^−/−^ rats (4.8 ± 0.2 mmol/L) compared to the Ctrl rats (5.5 ± 0.2 mmol/L) (*p* = 0.011). However, when comparing the two strains at each time point, a significant difference was only observed at baseline (*p* = 0.024), with lower levels in the *apoE*^−/−^ rats.

### *ApoE*^−/−^ rats develop systemic and hepatic hyperlipidemia

**Fig. 2:**
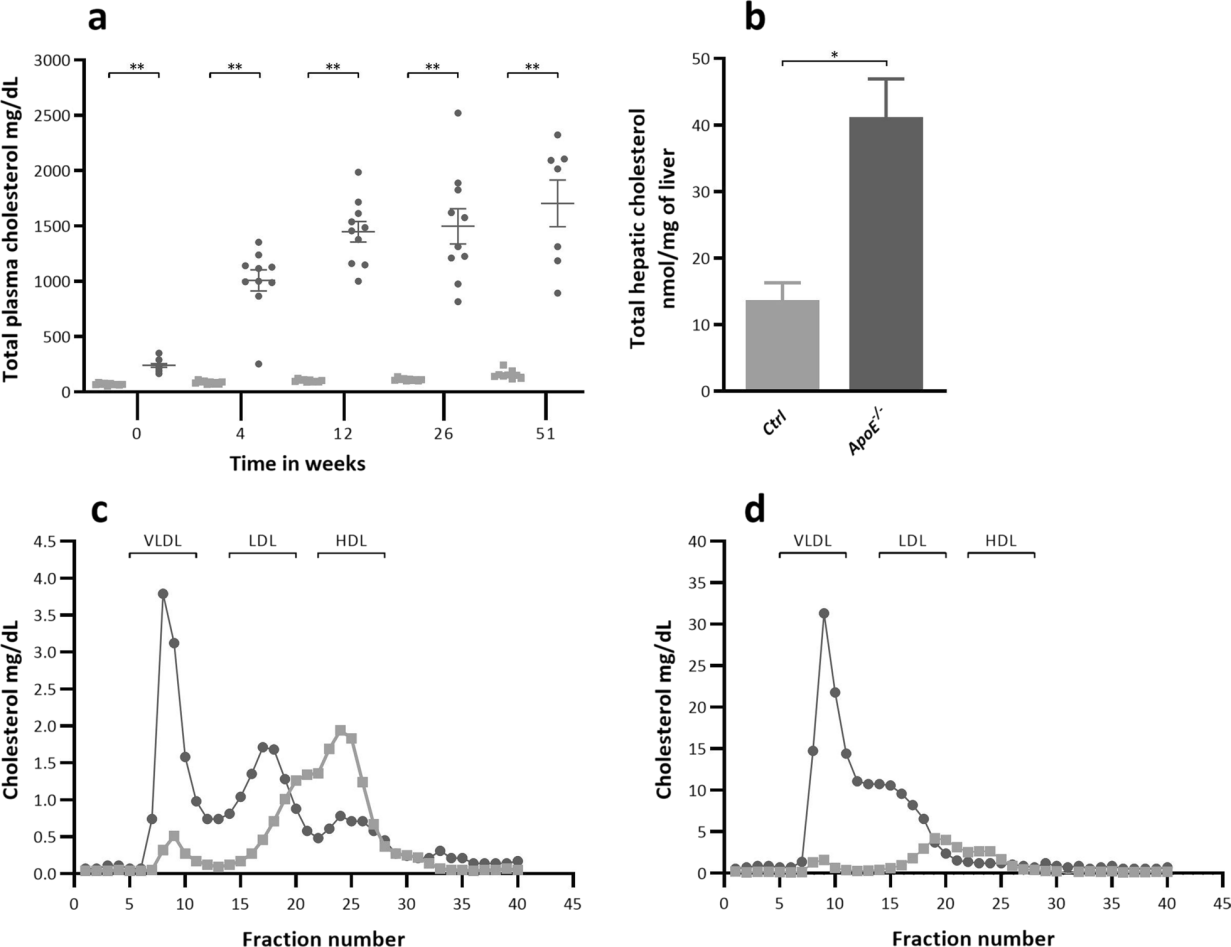
**a.** Total plasma cholesterol in mg/dl per strain and per time point. **b** Total hepatic cholesterol (nmol/mg of liver) at the end of the study at week 51 ± 1. Filled square Ctrl filled circle *ApoE*^*−/−*^. Data are presented as mean ± SEM. * is considered significantly different with a *p*-value < 0.05 and ** significantly different with a *p*-value < 0.001. **c** FPLC cholesterol profile from pooled plasma at baseline. **d** FPLC cholesterol profile from pooled plasma at the end of the study at week 51 ± 1

Total plasma cholesterol levels (Fig. 2a) were significantly higher in the *apoE*^−/−^ rats compared to the Ctrl rats from baseline to the end of the study (*p* < 0.001). Cholesterol levels increased in the *apoE*^−/−^ rats during the experiment, while they remained relatively stable in the Ctrl rats. After the start of the Western diet, cholesterol levels increased within 4 weeks from 240 ± 17 mg/dL to 1008 ± 90 mg/dL in the *apoE*^−/−^ rats and from 69 ± 3 to 86 ± 4 mg/dL in the Ctrl rats.

At the end of the study, total hepatic cholesterol levels were significantly higher in the *apoE*^−/−^ rats compared to the Ctrl rats (Fig. 2b) (*p* < 0.001).

The FPLC cholesterol profiles from pooled plasma at baseline (Fig. 2c) and at the end of the study (Fig. 2d) showed marked differences between the two genotypes. At baseline, the *apoE*^−/−^ rats had higher levels of very low-density lipoprotein (VLDL) and low-density lipoprotein (LDL) cholesterol compared to the Ctrl rats. Although a clear high-density lipoprotein (HDL) peak was still discernible in the *apoE*^−/−^ rat profile, it was lower than in the Ctrl rat profile. At the end of the study, a similar trend was observed in the cholesterol profile for the *apoE*^−/−^ rats, but the differences were more pronounced, with almost 10-folds higher levels, mainly in the pro-atherogenic apoB-containing VLDL and LDL fractions.

Overall plasma triglycerides were significantly higher (*p* < 0.001) in the *apoE*^−/−^ rats (506 ± 66 mg/dl) compared to Ctrl rats (344 ± 21 mg/dl). Per time point this difference was only noted at baseline (*p* < 0.001) and at the end of the study (Table 1). Total hepatic triglycerides were significantly higher in the *apoE*^−/−^ rats (*p* < 0.001).

**Table 1:** Total plasma and total hepatic triglycerides

	**Ctrl**	*apoE*^*−/−*^
*Total plasma triglycerides*		
mg/dL		
Baseline	156 ± 10	379 ± 41**
4 wks	582 ± 50	499 ± 35
12 wks	327 ± 40	355 ± 26
26 wks	363 ± 35	455 ± 55
51 wks	292 ± 40.0	842 ± 218*
*Total hepatic triglycerides*		
nmol/mg liver		
51 wks	16.4 ± 7.5	53.5 ± 15.2**

Data are presented as mean ± SD

*Significantly different with a *p*-value < 0.05

**Significantly different with a *p*-value < 0.001

Total cecal bile acid levels were significantly higher in the *apoE*^−/−^ rats compared to the Ctrl rats (*p* = 0.002, Table 2). This increase in total bile acids was primarily driven by elevated levels of lithocholic acid (*p* = 0.005), α-muricholic acid (*p* = 0.001), deoxycholic acid (*p* = 0.02), and chenodeoxycholic acid (*p* = 0.04).

**Table 2:** Fecal neutral sterols and fecal bile acids from cecum collected at the end of the study

	Ctrl	*apoE*^*−/−*^
*Fecal bile acids*		
nmol/mg cecum content		
α-muricholic acid	1.70 ± 0.55	4.56 ± 1.70*
β-muricholic acid	9.17 ± 4.95	10.87 ± 6.44
ω-muricholic acid	3.38 ± 1.69	5.14 ± 4.16
Chenodeoxycholic acid	0.08 ± 0.11	0.41 ± 0.36*
Cholic acid	3.10 ± 4.49	9.36 ± 8.53
Deoxycholic acid	4.94 ± 2.70	15.05 ± 9.05*
Lithocholic acid	0.54 ± 0.14	0.98 ± 0.31*
Total	22.90 ± 8.81	46.37 ± 12.69*
*Fecal neutral sterols*		
nmol/mg cecum content		
Coprostanol	6.59 ± 2.35	8.76 ± 3.10
Cholesterol	12.26 ± 5.82	8.91 ± 5.31
Dihydrocholesterol	0.13 ± 0.02	0.16 ± 0.06
Total	18.98 ± 5.09	17.82 ± 5.76

Data are expressed as mean ± SD

*Considered significantly different with a *p* value < 0.05

No significant differences were observed in the levels of fecal neutral sterols between the two strains.

### Higher [^18^F]FDG uptake in the aortic arch and abdominal aorta of the *apoE*^−/−^ rats

**Fig. 3:**
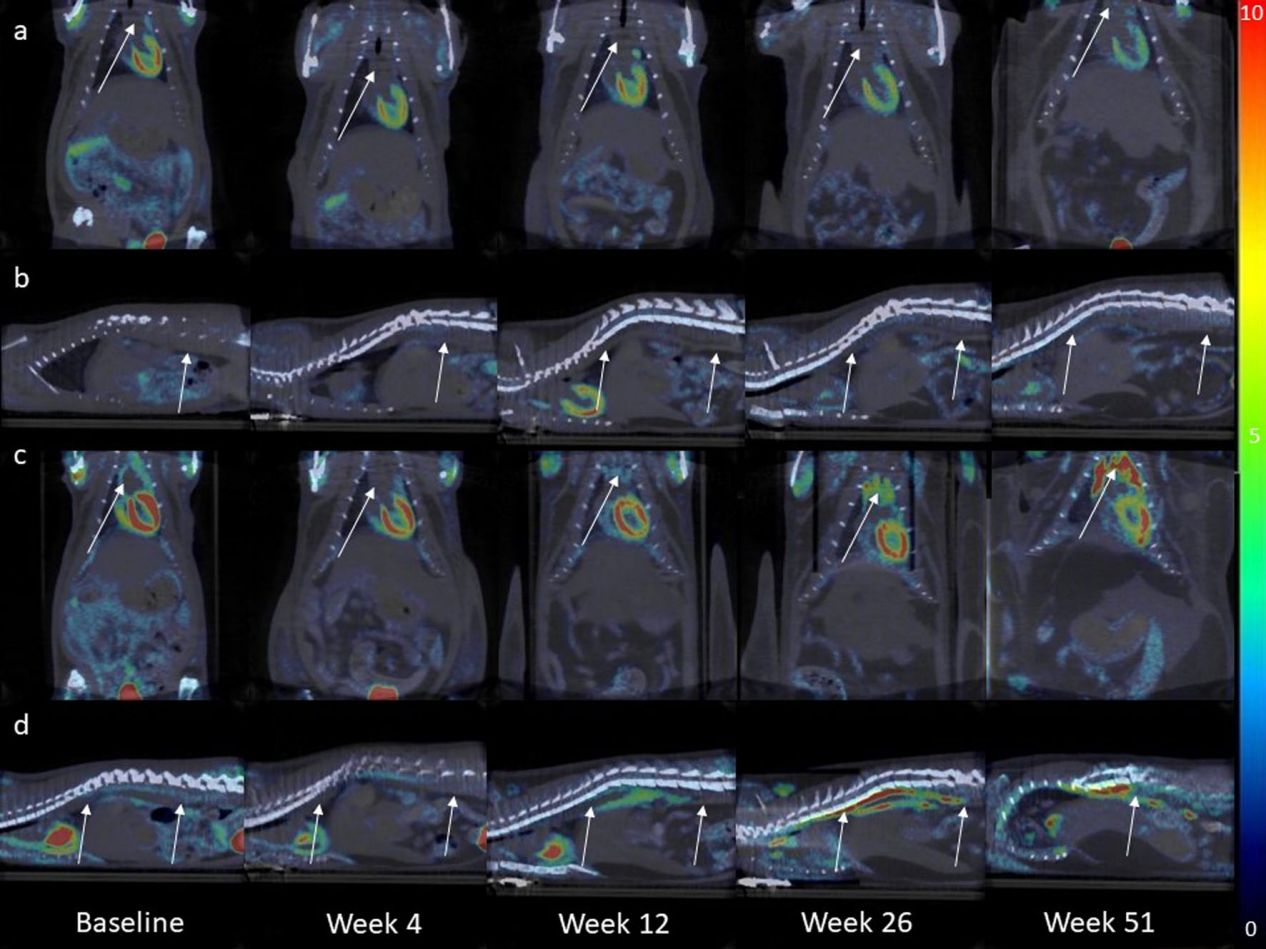
Representative fused [^18^F]FDG PET/CT images of a Ctrl and an *apoE*^*−/−*^ rat at different time points. (**a**) Shows a coronal view, and (**b**) presents a sagittal view of the same Ctrl rat. Similarly, (**c**) illustrates a coronal view, and (**d**) displays a sagittal view of the same *apoE*^-/-^ rat. The arrows indicate either the aortic arch or the abdominal aorta. Scalebar indicates SUV

**Fig. 4:**
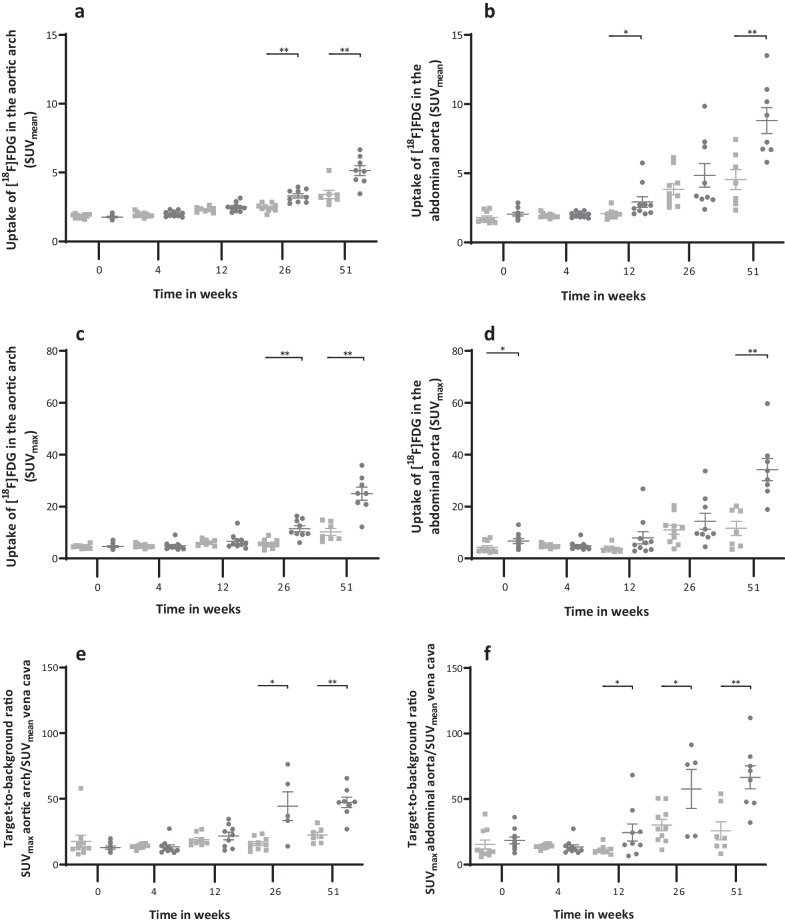
SUV_mean_ of the aortic arch (**a**) and the abdominal aorta (**b**). [^18^F]FDG uptake (SUV_max_) in the aortic arch (**c**) and the abdominal aorta (**d**) per strain per time point. Target-to-background ratio of the aortic arch (**e**) and the abdominal aorta (**f**). Graphs show the values per strain per time point. Filled square Ctrl filled circle *ApoE*^*−/−*^. Data are presented as mean ± SEM. * is considered significantly different with a *p*-value < 0.05 and ** significantly different with a *p*-value < 0.001

In the aortic arch (Fig. 3), the standard uptake value (SUV_mean_) of [^18^F]FDG was 18.5% higher (*p* < 0.001) in *apoE*^−/−^ rats (2.9 ± 0.1) compared to Ctrl rats (2.4 ± 0.08) (Fig. 4a). The difference in SUV_mean_ between the two groups was first observed at week 26 (2.5 ± 0.1 *vs* 3.3 ± 0.1, *p* < 0.001) and persisted until week 51 (3.4 ± 0.3 vs 5.1 ± 0.34, *p* < 0.001).

Similarly, in the abdominal aorta (Fig. 4b), the SUV_mean_ of [^18^F]FDG was 31.0% higher (*p* < 0.001) in *apoE*^−/−^ rats (4.1 ± 0.3) compared to Ctrl rats (2.9 ± 0.2). The difference in SUV_mean_ between the two groups was first observed at week 12 (2.1 ± 0.1 vs 2.9 ± 0.4, *p* = 0.024) and remained significantly higher up to week 51 (4.6 ± 0.7 vs 8.8 ± 0.9, *p* < 0.001).

When examining the temporal effects, both groups showed an increase in [^18^F]FDG uptake over time. The increase was more pronounced in the *apoE*^−/−^ group, with 4.3-folds higher SUVs compared to baseline, while the Ctrl group exhibited a 2.5-folds higher SUV levels.

The maximum [^18^F]FDG uptake (SUV_max_) in the aortic arch (Fig. 4c) and the abdominal aorta (Fig. 4d) was also significantly higher in *apoE*^−/−^ rats compared to Ctrl rats (*p* < 0.001). Since high plasma glucose levels can reduce [^18^F]FDG uptake we corrected uptake for plasma glucose levels (SUV_corr_). Notably, differences in uptake between the groups were observed in the aortic arch (*p* < 0.001) but not in the abdominal aorta (Additional file 3: Figure S1.a and b).

TBR (Fig. 4e–f) was significantly higher in the *apoE*^−/−^ rats at week 26 (*p* = 0.004) and week 51 (*p* < 0.001) in the aortic arch, and at weeks 12 (*p* = 0.037), 26 (*p* = 0.048), and 51 (*p* < 0.001) in the abdominal aorta.

**Fig. 5:**
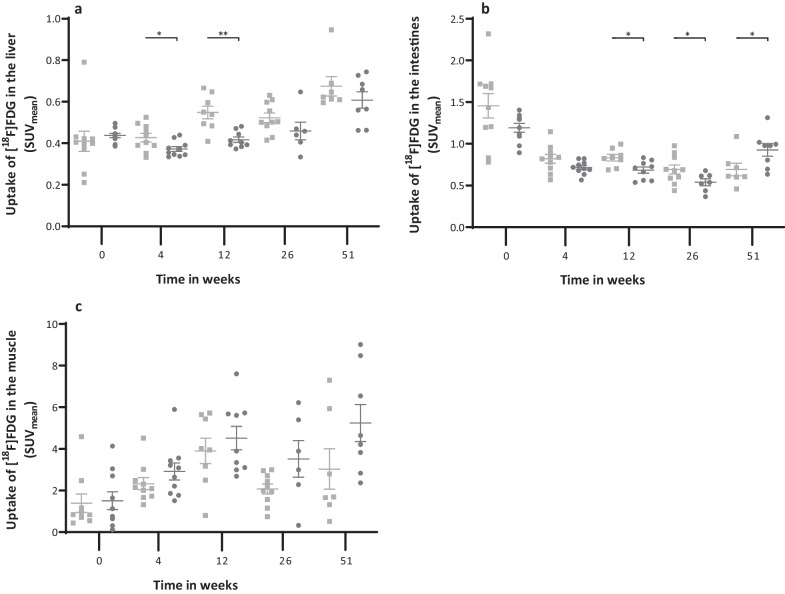
SUV_mean_ of the liver (**a**), intestines (**b**) and muscle (**c**). Graphs show the values per strain per time point. Filled square Ctrl filled circle *ApoE*^*−/−*^. Data is presented as mean ± SEM. * is considered significantly different with a *p*-value < 0.05 and ** significantly different with a *p*-value < 0.001

[^18^F]FDG uptake in the liver (Fig. 5a) was 11.1% lower (*p* < 0.011) in the *apoE*^−/−^ rats. This difference in uptake between the two groups was observed at weeks 4 (*p* = 0.014) and 12 (*p* < 0.001). A similar pattern was observed in the intestines (Fig. 5b) with a 10.2% (*p* = 0.036) lower uptake at weeks 12 (*p* = 0.003), 26 (*p* = 0.005), and this persisted until week 51 (*p* = 0.018). Uptake in the muscle (Fig. 5c) was overall 28.1% higher in the *apoE*^−/−^ rats (*p* = 0.012), but this could not be attributed to a specific time point. No strain effects were observed in the lungs, kidneys, vena cava, myocardium, and blood pool (Additional file 3: Figure S1.c-g).

### Lipid deposits, atherosclerotic lesions and macrophage accumulation reveal early plaque formation

**Fig. 6:**
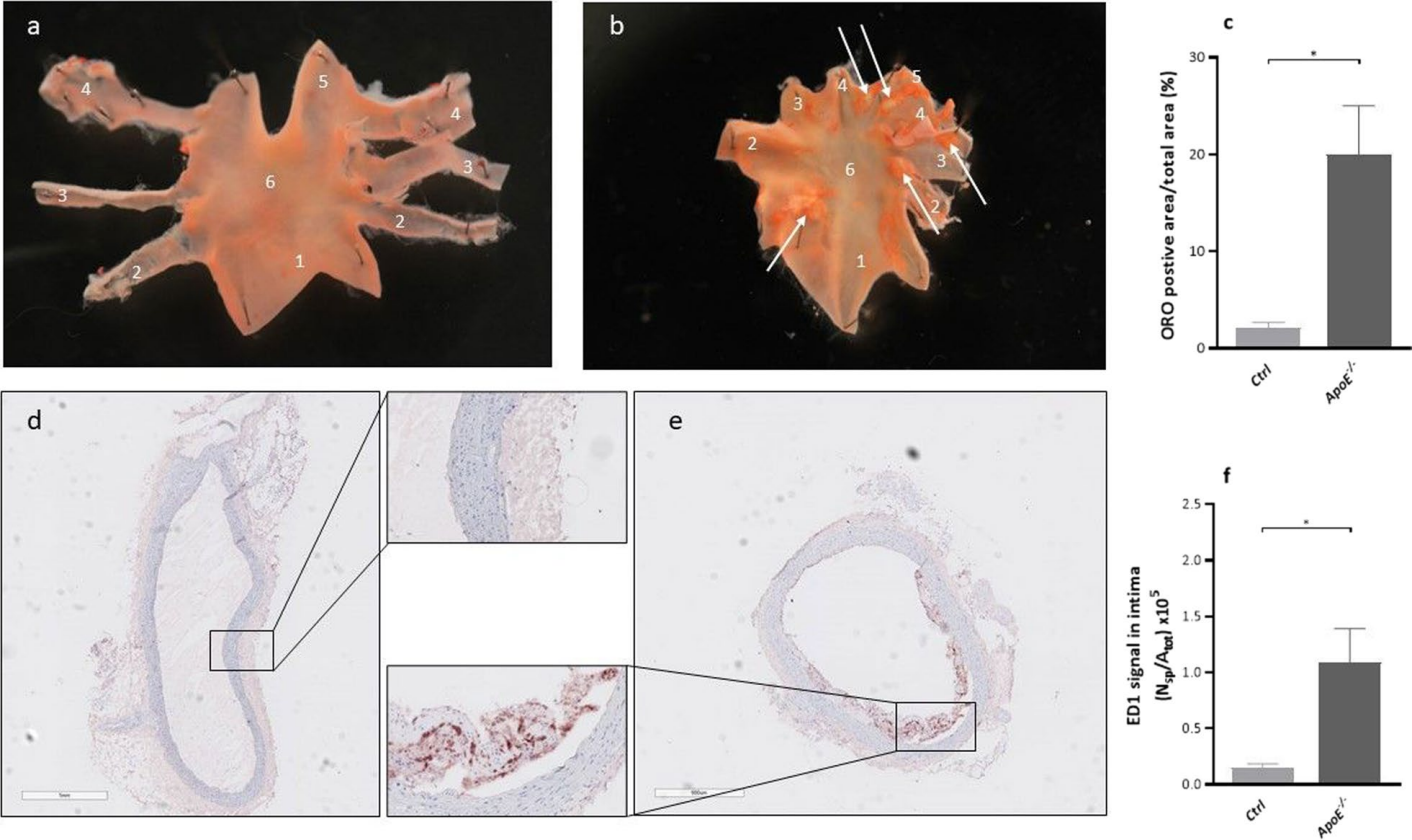
Representative images of ORO-stained aortic arches from an Ctrl rat (**a**) and an *apoE*^*−/−*^ rat (**b**), respectively, after treatment of 51 ± 1 weeks with a Western diet. The arch of the *apoE*^*−/−*^ rat is less uniform with more intense red areas (indicated by the white arrows). 1, Ascending aorta 2, Brachiocephalic artery, 3, Left common carotid artery, 4, Left subclavian artery, 5, Descending aorta, 6, Aortic arch. (**c**) Lipid-loaden atherosclerotic plaques presented as ORO positive area/total arch area. Representative images of ED1 staining (red) in the abdominal aorta from an Ctrl rat (**d**) and an *apoE*^*−/−*^ rat (**e**). Inserts represent a magnification (×20) of highlighted sections. (**f**) Quantification of the ED1 positive signal in the aortic intima presented as number of strong positive pixels/total surface (N_sp_/A_tot_). Data are presented as mean ± SEM. * is considered significantly different with a *p*-value < 0.05

Neutral lipid deposits were clearly visible in the *apoE*^−/−^ rats when staining the aortic arch with Oil Red O (ORO). The staining (Fig. 6a and b) in *apoE*^−/−^ rats showed signs of atherosclerotic lesions which covered (20 ± 13% of the surface of the intima, whereas only 2 ± 1% of the intima surface stained positive for ORO in the Ctrl rats (Fig. 6c, p = 0.001).

In the *apoE*^−/−^ rats, there was an accumulation of ED1-positive macrophages (Fig. 6d, e, f), indicating increased macrophage accumulation in the subendothelial space of the abdominal aorta (*p* = 0.010). In contrast, the Ctrl rats showed minimal presence of ED1-positive macrophages.

**Fig. 7:**
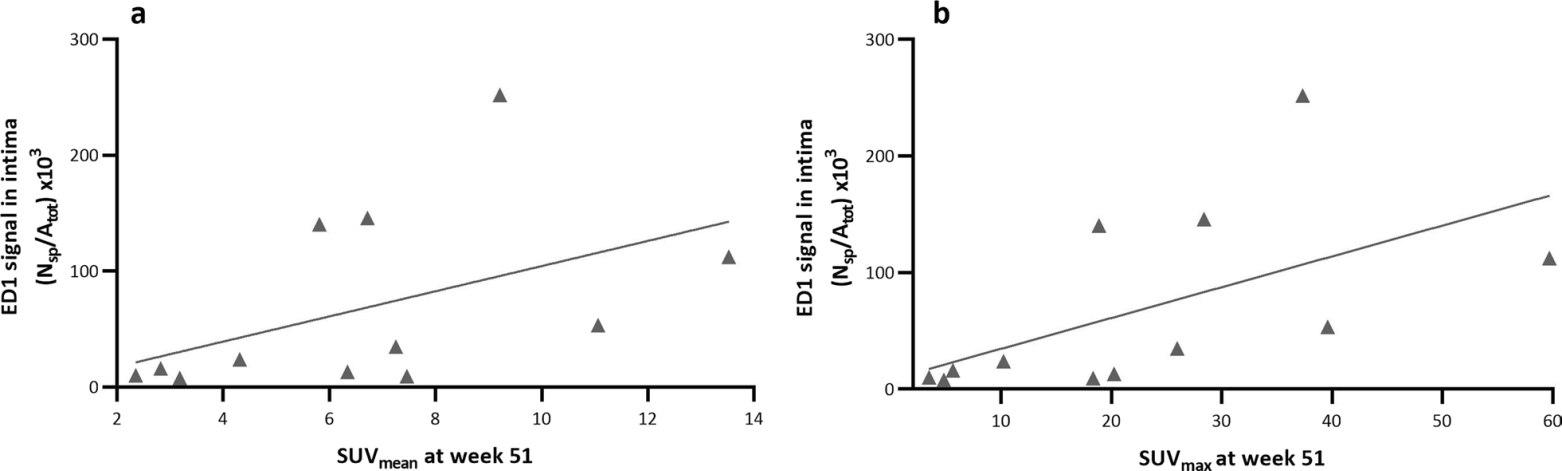
**a.** Correlation between average uptake of [^18^F]FDG and ED1-positive macrophages in the abdominal aorta (*r* = 0.5, *p* = 0.061). (**b**) Correlation between maximum uptake of [^18^F]FDG and ED1 positive macrophages in the abdominal aorta (*r* = 0.6, *p* = 0.027)

A weak correlation (*r* = 0.5) (Fig. 7a) between SUV_mean_ in the abdominal aorta at week 51 and the number of ED1-positive macrophages was found but it did not reach statistical significance (*p* = 0.061). However, a correlation was observed between SUV_max_ and the number of macrophages (Fig. 7b). The correlation coefficient (*r*) was 0.6, indicating a moderate positive correlation between the two variables (*p* = 0.027). Additionally, a correlation was observed between SUV and plasma cholesterol levels, SUV and ORO positive area, as well as plasma cholesterol levels and ORO positive area (Additional file 4: Figure S2.a–g).

## Discussion

This study establishes the *apoE*^−/−^ rat as suitable model for atherosclerosis imaging.

The phenotype observed in the *apoE*^−/−^ rats used in our study aligns with data from previous studies conducted by our institute and others. Using the same model, nearly all studies have reported signs of hypercholesterolemia, characterized by elevated levels of total plasma cholesterol and total plasma triglycerides, after 8 weeks of feeding an atherogenic diet [14–16, 20, 25–27]. Some studies have noted the absence of lesions in different regions of the aorta [14, 15, 25], which can be attributed to the relatively short duration of treatment with an atherogenic diet and the young to medium age of the rats. Other studies have observed lesions, but only after introducing a secondary trigger such as occlusal disharmony [26] or challenging endothelial injury [14]. Gao et al*.* utilized a Paigen (high cholesterol/bile salt) diet for 10–12 weeks and observed severe coronary atherosclerosis characterized by significant lipid accumulation, macrophage accumulation, and collagen fibers deposits. However, the authors noted only mild atherosclerosis in the aortic root and over the full length of the aorta [16].

In our previous work, which focused on the modulation of aortic relaxation by perivascular adipose tissue (PVAT), we concluded that the *apoE*^−/−^ rat serves as a model for early-stage atherosclerosis. The data revealed an increased intima/media thickness ratio and an influx of ED1-positive macrophages in the aortic intima of *apoE*^−/−^ rats. Consistent with the findings of this study, we observed deposits of plaque formation in the thoracic aortas of *apoE*^−/−^ rats [20].

When comparing the phenotype of *apoE*^−/−^ rats with *apoE*^−/−^ mice, similar results were observed. Zhou et al*.* reported hypercholesterolemia in mice aged 40–52 weeks treated with an atherogenic diet for 8 weeks [28]. Similarly, Gogulamudi et al*.* reported similar levels of cholesterol and triglycerides in 19-month-old *apoE*^−/−^ mice fed an atherogenic diet [29]. Despite the comparable results in cholesterol and triglyceride levels, the severity and composition of the plaques in mice were significantly different. Several studies reported high plaque burden (around 20%) [29, 30], and severe atheromas with necrotic cores and collagen deposition in the media in old *apoE*^−/−^ mice [29]. In young mice (15 weeks of age), intermediate lesions containing foam cells and smooth muscle cells were found, and after 20 weeks, fibrous plaques appeared [4]. The difference in plaque severity and composition between *apoE*^−/−^ mice and rats is not yet fully understood but could be explained by species differences [25] or by the background strain of the rats, which may be more resistant to atherosclerosis [27].

The results of this study were based on male rats only. Data from the supplier and previous studies in *apoE*^−/−^ mice and rats showed significantly elevated plasma cholesterol levels in males compared to females [31], or a tendency for higher levels [32]. Kong et al*.* suggested that the differences between the two sexes disappeared after females reached menopause [31], which suggests a potential effect of estrogen on plasma cholesterol. For future studies using this model, we recommend including both male and female rats to facilitate scientific and therapeutic discoveries for both sexes [33].

The PET data in our study are consistent with the findings previously described by Zhuang et al*.* [34]. Although the SUV_mean_ and SUV_max_ were higher in our study, the differences in [^18^F]FDG uptake between control and knockout rats, as well as the time points when the differences occurred, were comparable. However, despite the similarity in [^18^F]FDG data between the two studies, the conclusions drawn are quite different. Zhuang et al*.* concluded that [^18^F]FDG uptake in the aortic arch is associated with HIF-1α gene expression (hypoxia) rather than inflammatory lesions. The authors observed high expression of HIF-1α in the aortic arch, which exhibited high [^18^F]FDG uptake. Conversely, they noticed the opposite effect in the pulmonary arteries, with high CD68 expression but lower uptake of [^18^F]FDG [34]. Although no clear correlation data were presented, their conclusion was supported by Laurberg et al., who did not find [^18^F]FDG accumulation in advanced atherosclerotic lesions of *apoE*^−/−^ mice [13], and Myers et al., who did not find a correlation between [^18^F]FDG and CD68 expression [35]. An explanation for the disparities between Zhuang et al. and our study could stem from variances in the genetic models employed (Biocytogen vs Sage Labs Inc.) or the varying compositions of the diets utilized (42% fat vs 21% fat). An alternative explanation could be the chosen imaging procedure. It has been suggested in different studies to perform [^18^F]FDG PET imaging 3 h after injection to maximize the contrast between plaque and background [17–19, 36, 37]. In our study, we observed a weak-to-moderate correlation between [^18^F]FDG uptake and ED1-positive cells. The correlation was lower than reported by Tawakol et al*.* [19] which could be explained by the rather poor co-registration of the PET imaging with the immunohistochemistry images. In this study, we quantified the number of macrophages in a random section of the abdominal aorta between the diaphragm and hepatic and splenic arteries. For [^18^F]FDG uptake, we used a volume of interest (VOI) measuring 15 × 15 × 15 mm positioned in line with the diaphragm. Despite the limited overlap, we found a correlation between [^18^F]FDG uptake and the ED1 signal, which is consistent with findings from various studies in patients and animals [8, 18, 37, 38]. An alternative explanation for the observed lower correlation may stem from the timing of the conducted immunohistochemistry. Silvola et al. have recommended utilizing LDLR^*−/−*^ApoB and IGF-II/LDLR^*−/−*^ApoB mice that are at least 6 months old prior to imaging. This recommendation is based on their observation of highly inflamed, large, and extensive atherosclerotic plaques with the highest FDG uptake occurring in plaques characterized by a high macrophage density [39]. It is conceivable that the time point of 51 ± 1 weeks might not be optimal for identifying plaques with a high macrophage density in *apoE*^−/−^ rats. Considering the relatively slow plaque development in our study, it is plausible that the opportune time point for such plaques may fall later than the 52-week mark.

Another noteworthy detail in our data is the increasing [^18^F]FDG uptake over time observed in the control rats. This effect has been reported previously [40] and is most likely associated with the advanced age of the control rats combined with the Western diet [29].

For the PET analysis, we selected two volumes of interest (VOIs) located on different sections of the aorta. The aortic arch was chosen due to its curvature and the presence of various bifurcations, which cause disturbed shear stress. Disturbed shear stress is known to play a role in the pathophysiology of atherosclerosis and is associated with atheroma formation near bifurcations and curvatures [41]. The second VOI was positioned on the abdominal aorta directly below the diaphragm. Although this section lacks a curve, it is rich in bifurcations. Additionally, the distance between the heart and this part of the aorta is sufficient to avoid spillover effects.

In this study, we exclusively used [^18^F]FDG as the PET tracer. The choice of [^18^F]FDG was based on its association with vascular inflammation [42, 43], the extensive use of [^18^F]FDG PET imaging in atherosclerosis studies, and its status as the most commonly used and clinically available PET tracer. Imaging with other tracers such as ^18^F-Sodium Fluoride (micro-calcification) [44] and ^11^C/^18^F-choline (for macrophages in atherosclerotic plaques) [45] could yield different conclusions due to variations in uptake pathophysiology.

Bile acids and neutral sterols play a significant role in the development of dyslipidemia. Breuninger et al*.* reported positive associations between fecal bile acids and markers of dyslipidemia, as well as between fecal cholesterol and hypertriglyceridemia [46]. In our study, we did not observe a significant difference in neutral sterols between the two strains, but higher levels of bile acids were found in *apoE*^−/−^ rats, suggesting that the disparity in lipid metabolism in *apoE*^−/−^ rats extends to bile acids.

## Limitations

Due to technical issues with sample preservation and preparations, we were limited to ORO staining in the aortic arch and ED1 staining in the abdominal aorta only. Additional histological analyses (smooth muscle cell activation, endothelial cells, fibroblasts, microcalcification) in the aortic tissue or immunohistochemistry in other tissues would have provided a more comprehensive characterization of the model.

For this study, we based our scanning procedure partly on the suggestion by Rudd et al. [36]. We are aware that the choices in the procedure (non-fasting, isoflurane anesthesia, interval between injection and scan) could have an impact on the physiological uptake of [^18^F]FDG. It is therefore possible that the chosen protocol may not be optimal for atherosclerotic imaging in this model. We suggest an additional study to explore the best scan procedure for [^18^F]FDG PET/CT imaging of atherosclerosis in this model.

Secondly, it can be debated that the time points in this study are not the best moments to image inflammation in atherosclerotic plaques. To address this, we suggest an additional study with histological analysis at each imaging time point, alongside an assessment of the correlation between [^18^F]FDG signal and inflammation.

Although the *apoE*^−/−^ rat is a promising model for atherosclerotic PET/CT imaging, the model still has some disadvantages which were also observed in *apoE*^−/−^ mice. Therefore, it would be of great interest to explore other genetic models for atherosclerosis, like the *LDLR*^−/−^ rat model.

Our studies show that the *apoE*^−/−^ rat is a viable model for longitudinal atherosclerotic PET imaging. This model exhibits hypercholesterolemia, increased bile acids and triglycerides, leading to the formation of early atherosclerotic lesions accompanied by macrophage accumulation. The gradual progression of the disease in this model resembles the rather slow development of atherosclerosis in the human condition, making it particularly suitable for investigating early stages of atherosclerosis in a non-invasive manner.

### Supplementary Information

**Additional file 1. Table S1**. Content of Western diet D12079B (Research Diets Inc. New Brunswick, New Jersey, United States). *Anhydrous milk fat typically contains approximately 0.3% cholesterol. On this basis, D12079B contains approximately 0.21% cholesterol.

**Additional file 2. Table S2**. The quantification of macrophages in the ED-1 immunohistochemistry staining using Aperio ImageScope software (version v12.4.3.5008, Leica Biosystems Imaging Inc., Vista, CA, U.S.A.) was performed using the following algorithm.

**Additional file 3. Figure S1**. [^18^F]FDG uptake corrected for plasma glucose levels (SUV_corr_). in the the aortic arch (**a**) and the abdominal aorta (**b**) per strain per time point. Uptake was normalized for glucose levels at the time of tracer injection using SUV_corr_ = SUV * glucose levels / glucose_group average_. SUV_mean_ of the lungs (**c**), kidneys (**d**), vena cava (**e**), myocardium (**f**) and blood pool (**g**). Filled square Ctrl filled circle *ApoE*^−/−^. Data is presented as mean ± SEM. * is considered significantly different with a *p*-value < 0.05 and ** significantly different with a *p*-value < 0.001.

**Additional file 4.** (**a**) Correlation between SUV_mean_ in the aortic arch and total plamsa cholesterol, (**b**) correlation between SUV_max_ in the aortic arch and total plamsa cholesterol, (**c**) correlation between SUV_mean_ in the abdominal aorta and total plamsa cholesterol, (**d**) correlation between SUV_max_ in the aortic arch and total plamsa cholesterol, (**e**) correlation between SUV_mean_ in the aortic arch and ORO positive area, (**f**) correlation between SUV_max_ in the aortic arch and ORO positive area, (**g**) correlation between ORO positive area and total plamsa cholesterol. Filled square Ctrl filled circle *ApoE*^−/−^.

## Data Availability

Data reported in this paper are archived in the University Medical Center Groningen, University of Groningen and are available on request.

## Abbreviations

PET: Positron emission tomography
CT: Computed tomography
aopE: Apolipoprotein E
*apoE*^*−*^^*/*^^*−*^: Apolipoprotein E-deficient
Ctrl: Control surplus rats (Sprague Dawley)
[^18^F]FDG: [^18^F]2-fluoro-2-deoxy-D-glucose
VLDL: Very low–density lipoprotein
LDL: Low–density lipoprotein
HDL: High–density lipoprotein
SUV: Standardized uptake value
GEE: Generalized estimating equations
VOI: Volume of interest
ROI: Region of interest
TBR: Target-to-background ratio
OSEM3D/MAP: Ordered subset expectation maximization 3D/maximum a posteriori
FPLC: Fast protein liquid chromatography
