# Supplementary material for: Characterization of a novel model for atherosclerosis imaging: the apolipoprotein E-deficient rat
Source: EJNMMI Res. 2023 Dec 11;13:106. doi: 10.1186/s13550-023-01055-5 (PMC10713960; doi:10.1186/s13550-023-01055-5)
Supplement: Supplementary file 1 — Additional file 1. Table S1. Content of Western diet D12079B (Research Diets Inc. New Brunswick, New Jersey, United States). *Anhydrous milk fat typically contains approximately 0.3% cholesterol. On this basis, D12079B contains approximately 0.21% cholesterol. [file 13550_2023_1055_MOESM1_ESM.docx]

***Supplementary table S1.*** *Content of* *Western diet D12079B (Research Diets Inc. New Brunswick, New Jersey, United States). *Anhydrous milk fat typically contains approximately 0.3% cholesterol. On this basis, D12079B contains approximately 0.21% cholesterol.*

| **Diet D12079B** |  |  |  |
| --- | --- | --- | --- |
|  |  | **gm%** | **kcal%** |
|  |  |  |  |
| **Protein** |  | 20 | 17 |
| **Carbohydrate** |  | 50 | 43 |
| **Fat** |  | 21 | 41 |
|  | **Kcal/gm** | 4.7 |  |
|  | **Total** |  | 100 |
|  |  |  |  |
| **Ingredient** |  | **gm** | **kcal** |
| **Casein 80 Mesh** |  | 195 | 780 |
| **DL-Methionine** |  | 3 | 12 |
| **Corn Starch** |  | 50 | 200 |
| **Maltodextrin 10** |  | 100 | 400 |
| **Sucrose** |  | 341 | 1364 |
| **Cellulose** |  | 50 | 0 |
| **Milk fat*** |  | 200 | 1800 |
| **Corn Oil** |  | 10 | 90 |
| **Mineral Mix S10001** |  | 35 | 0 |
| **Calcium Carbonate** |  | 4 | 0 |
| **Vitamine Mix V10001** |  | 10 | 40 |
| **Choline Bitartrate** |  | 2 | 0 |
| **Cholesterol** |  | 1.5 | 0 |
| **Ethoxyquin** |  | 0.04 | 0 |
|  |  |  |  |
|  |  |  |  |
| **Total** |  | **1001.54** | **4686** |
