# Supplementary material for: Characterization of a novel model for atherosclerosis imaging: the apolipoprotein E-deficient rat
Source: EJNMMI Res. 2023 Dec 11;13:106. doi: 10.1186/s13550-023-01055-5 (PMC10713960; doi:10.1186/s13550-023-01055-5)
Supplement: Supplementary file 2 — Additional file 2. Table S2. The quantification of macrophages in the ED-1 immunohistochemistry staining using Aperio ImageScope software (version v12.4.3.5008, Leica Biosystems Imaging Inc., Vista, CA, U.S.A.) was performed using the following algorithm. [file 13550_2023_1055_MOESM2_ESM.docx]

***Supplementary table S2.*** *The quantification of macrophages in the ED-1 immunohistochemistry staining using Aperio ImageScope software (version v12.4.3.5008, Leica Biosystems Imaging Inc., Vista, CA, U.S.A.) was performed using the following algorithm:*

| **Specification** | **Value** |
| --- | --- |
| **View Width** | 1000 |
| **View Height** | 1000 |
| **Overlap Size** | 0 |
| **Image Zoom** | **1** |
| **Markup Compression Type** | Same as processed image |
| **Compression quality** | 30 |
| **Classifier Neighborhood** | 0 |
| **Classifier** | None |
| **Class List** |  |
| **Hue Value** | 1 |
| **Hue Width** | 0.4 |
| **Color Saturation Threshold** | 0.04 |
| **Iwp (High)** | 220 |
| **Ipw (Low) = Ip (High)** | 175 |
| **Ip (Low) = Isp (High)** | 130 |
| **Isp (Low)** | 0 |
| **Inp (High)** | -1 |
