# Supplementary figures and images for: Characterization of a novel model for atherosclerosis imaging: the apolipoprotein E-deficient rat
Source: EJNMMI Res. 2023 Dec 11;13:106. doi: 10.1186/s13550-023-01055-5 (PMC10713960; doi:10.1186/s13550-023-01055-5)

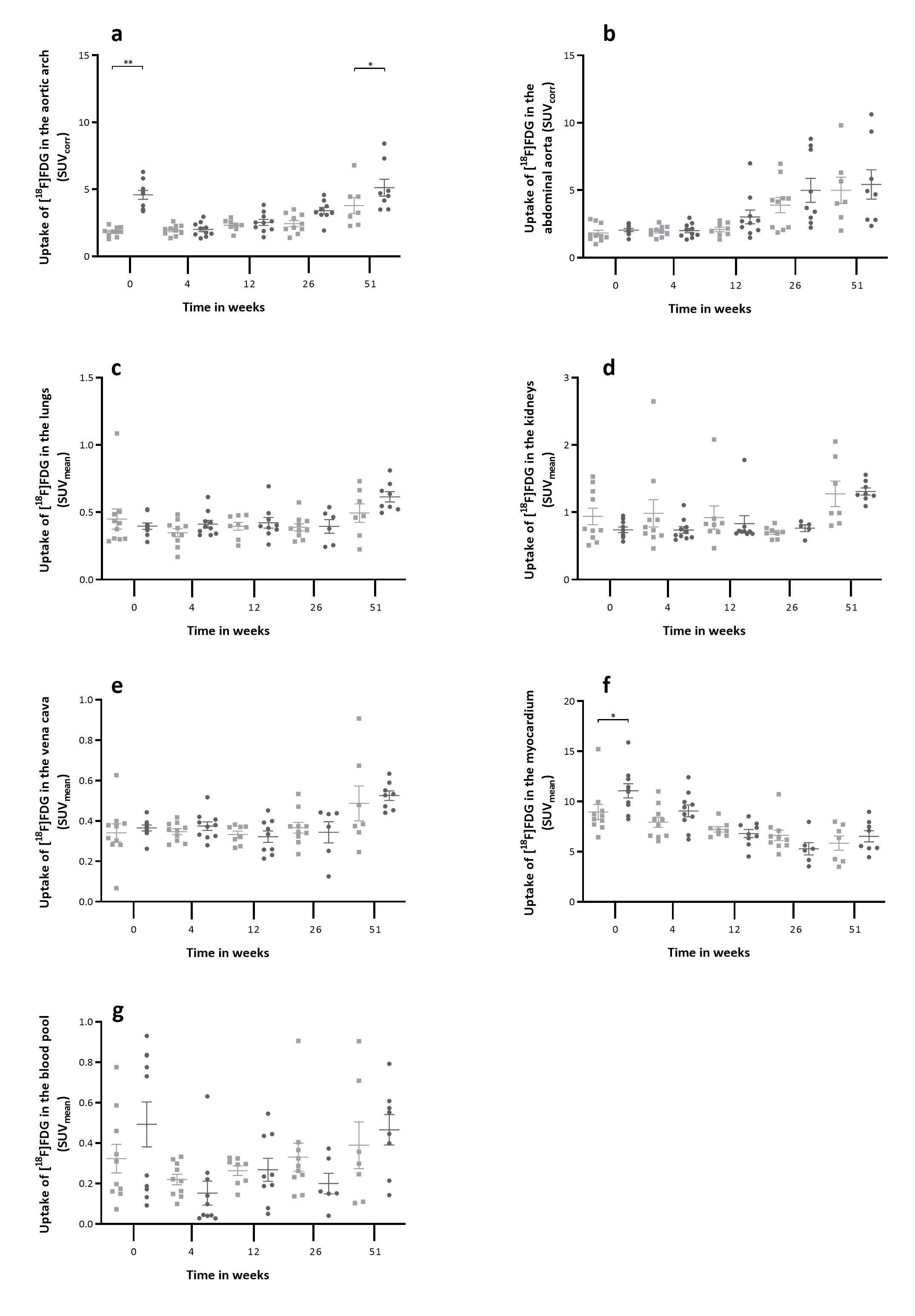

Supplement: Supplementary file 3 — Additional file 3. Figure S1. [18F]FDG uptake corrected for plasma glucose levels (SUVcorr). in the the aortic arch (a) and the abdominal aorta (b) per strain per time point. Uptake was normalized for glucose levels at the time of tracer injection using SUVcorr = SUV * glucose levels / glucosegroup average. SUVmean of the lungs (c), kidneys (d), vena cava (e), myocardium (f) and blood pool (g). Filled square Ctrl filled circle ApoE−/−. Data is presented as mean ± SEM. * is considered significantly different with a p-value < 0.05 and ** significantly different with a p-value < 0.001. [file 13550_2023_1055_MOESM3_ESM.jpg]

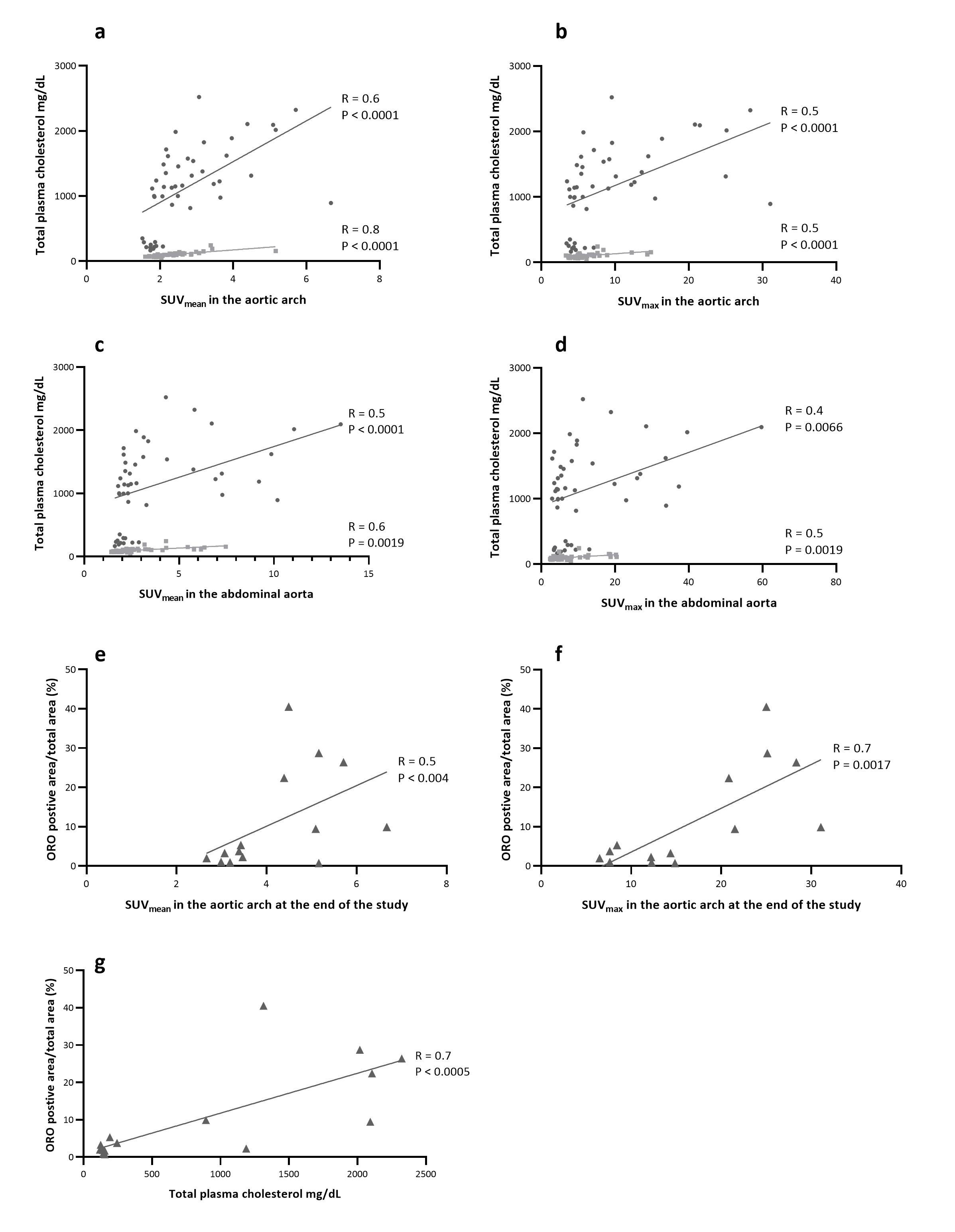

Supplement: Supplementary file 4 — Additional file 4. (a) Correlation between SUVmean in the aortic arch and total plamsa cholesterol, (b) correlation between SUVmax in the aortic arch and total plamsa cholesterol, (c) correlation between SUVmean in the abdominal aorta and total plamsa cholesterol, (d) correlation between SUVmax in the aortic arch and total plamsa cholesterol, (e) correlation between SUVmean in the aortic arch and ORO positive area, (f) correlation between SUVmax in the aortic arch and ORO positive area, (g) correlation between ORO positive area and total plamsa cholesterol. Filled square Ctrl filled circle ApoE−/−. [file 13550_2023_1055_MOESM4_ESM.jpg]
